# EFEMP1 induces γ-secretase/Notch-mediated temozolomide resistance in glioblastoma

**DOI:** 10.18632/oncotarget.1620

**Published:** 2013-12-07

**Authors:** Lotte Hiddingh, Bakhos A. Tannous, Jian Teng, Bas Tops, Judith Jeuken, Esther Hulleman, Sandra H. Boots-Sprenger, W. Peter Vandertop, David P. Noske, Gertjan J.L. Kaspers, Pieter Wesseling, Thomas Wurdinger

**Affiliations:** ^1^ Department of Neurosurgery, VU University Medical Center, Amsterdam, The Netherlands; ^2^ Department of Pediatric Oncology, VU University Medical Center, Amsterdam, The Netherlands; ^3^ Neuro-oncology Research Group, Cancer Center Amsterdam, VU University Medical Center, Amsterdam, The Netherlands; ^4^ Department of Neurology, Massachusetts General Hospital and Harvard Medical School, Boston, MA, USA; ^5^ Department of Pathology, Radboud University Nijmegen Medical Centre, Nijmegen, The Netherlands; ^6^ Department of Pathology, VU University Medical Center, Amsterdam, The Netherlands; ^7^ Department of Pathology, PAMM, Eindhoven, The Netherlands

**Keywords:** Temozolomide resistance, glioblastoma, EFEMP1, γ-secretase, Notch, GSI

## Abstract

Glioblastoma is the most common malignant primary brain tumor. Temozolomide (TMZ) is the standard chemotherapeutic agent for this disease. However, intrinsic and acquired TMZ-resistance represents a major obstacle for this therapy. In order to identify factors involved in TMZ-resistance, we engineered different TMZ-resistant glioblastoma cell lines. Gene expression analysis demonstrated that EFEMP1, an extracellular matrix protein, is associated with TMZ-resistant phenotype. Silencing of EFEMP1 in glioblastoma cells resulted in decreased cell survival following TMZ treatment, whereas overexpression caused TMZ-resistance. EFEMP1 acts via multiple signaling pathways, including γ-secretase-mediated activation of the Notch pathway. We show that inhibition of γ-secretase by RO4929097 causes at least partial sensitization of glioblastoma cells to temozolomide in vitro and in vivo. In addition, we show that EFEMP1 expression levels correlate with survival in TMZ-treated glioblastoma patients. Altogether our results suggest EFEMP1 as a potential therapeutic target to overcome TMZ-resistance in glioblastoma.

## INTRODUCTION

Glioblastoma is the most common malignant primary brain tumor in humans. Outcome for glioblastoma patients is dismal, and carries a median survival of only 14 months [1]. Standard treatment consists of surgery (if possible), followed by radiotherapy and adjuvant temozolomide (TMZ) chemotherapy [1, 2]. Although the addition of TMZ to radiotherapy has resulted in an overall increase in survival of glioblastoma patients, therapy still fails in almost all glioblastoma patients due to incomplete tumor resection, and/or the apparent resistance of tumor cells to irradiation and TMZ. Some tumors are insensitive to TMZ already at diagnosis, whereas others may develop acquired TMZ-resistance during treatment. Therefore, TMZ-resistance represents a major obstacle in the treatment of this disease.

The cytotoxic effect of TMZ is mainly mediated through induction of the DNA adduct O6-methylguanine (O6M-G) resulting in activation of the mismatch repair (MMR) system, induction of DNA double strand breaks, and subsequent cell death [3,4]. The alkylation of the O6 position of guanine can be counteracted by the MGMT protein (O6-methylguanine DNA methyltransferase). It is widely accepted that hypermethylation of the promoter of the MGMT gene in the tumor tissue can predict sensitivity to TMZ [5–7], since hypermethylation prevents the expression of MGMT thereby sensitizing the cells to TMZ [8, 9]. The highly relevant role of MGMT in response to TMZ is confirmed by the increased sensitivity when combining TMZ with the competitive MGMT inhibitor O6-benzylguanine [10–12]. Also the MMR status can be important for TMZ sensitivity, as a functional MMR mechanism is required to induce double strand breaks, and subsequent cell cycle arrest and apoptosis [3, 4, 13]. Defects in MMR have been suggested to be involved especially in acquired TMZ-resistance [14–16]. Besides the canonical MGMT and MMR TMZ-resistance mechanisms it is likely that non-canonical mechanisms can also contribute to TMZ-resistance. Further insight into the underlying mechanisms of non-canonical TMZ-resistance mechanisms may not only allow for better prediction of treatment response, and thus to individualized therapy, but may also provide targets for counteracting TMZ-resistance.

EFEMP1 (Fibulin-3) is an extracellular matrix protein involved in tumor progression in several types of cancer [17–20]. In glioblastoma, EFEMP1 has been reported to stimulate tumor growth, invasion of tumor cells, and resistance to apoptosis [21, 22]. EFEMP1 can exert these tumor promoting effects through activation of the Notch signaling pathway [22], although EFEMP1 was also reported to activate EGFR and the downstream AKT/PI3K/mTor, and MAPK pathways [19, 23]. Activation of the Notch cascade has been previously implicated in TMZ-resistance in glioblastoma, and plays an essential role in determining cell fates such as differentiation, proliferation, and apoptosis [24–26]. Here we identify by gene expression profiling of both TMZ-sensitive and non-canonical TMZ-resistant glioblastoma cell lines that expression of EFEMP1 is associated with a TMZ-resistant phenotype. Furthermore, we show that EFEMP1-mediated TMZ-resistance is regulated – at least partly – through the Notch pathway.

## RESULTS

### EFEMP1 is overexpressed in TMZ-resistant glioblastoma cells

**Figure 1:**
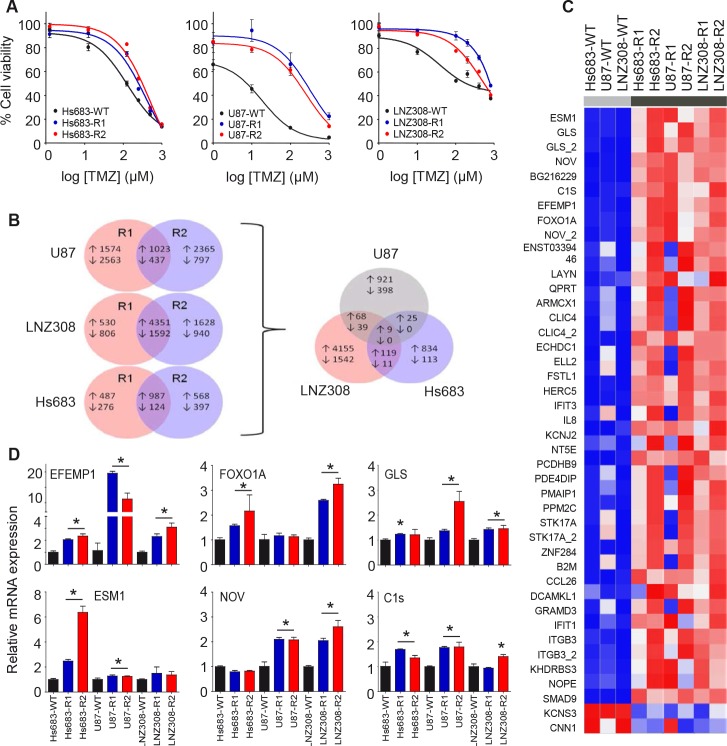
Identification of EFEMP1 as differentially expressed transcript in TMZ-resistant glioblastoma cells A, Hs683, U87, and LNZ308 glioblastoma cells (WT) and TMZ-resistant subclones (R1 and R2) were analyzed for TMZ sensitivity by automated cell counting at four days after TMZ treatment. B, number of differentially expressed transcripts overlapping between resistant subclones Rl and R2 of each individual glioblastoma cell line (left) and number of overlapping transcripts among the resistant glioblastoma subclones (right). C, transcripts that were differentially expressed in at least five out of six TMZ-resistant glioblastoma subclones are shown in heatmap format. D, the top-6 transcripts overexpressed in all six resistant subclones were validated by qRT-PCR. Shown are averages, error bars indicate SD. *p<0.05 t test.

In order to develop TMZ-resistant glioblastoma cells, we treated Hs683, U87, and LNZ308 glioblastoma cells twice a week with 33 μM TMZ for several weeks, resulting in two independent stable TMZ-resistant subclones for each glioblastoma cell line. The TMZ sensitivity was determined by automated cell counting at four days post-TMZ treatment (Fig. 1A). The IC50 values of the resistant glioblastoma subclones showed >2-fold increase in TMZ-resistance compared to the parental cell lines (Supplementary Table S1). These cell lines were characterized for MGMT methylation and MMR status to assess canonical TMZ-resistance mechanisms, but no significant differences were observed between the parental cell line and the resistant subclones (Supplementary Table S1), suggesting that a non-canonical TMZ-resistance mechanism was acquired by these cells, and – consequently – resulting in a useful tool to study non-canonical TMZ-resistance.

In order to determine which non-canonical mechanisms are responsible for the observed TMZ-resistance, we isolated RNA from the parental glioblastoma cells (designated WT) and the two independent TMZ-resistant subclones (designated R1 and R2). We performed gene expression array analysis and a significance analysis of microarrays (SAM analysis) using a false discovery rate of <10%. We identified transcripts that were differentially expressed between the WT and R1/R2 glioblastoma cells of each individual glioblastoma cell line and overlapping transcripts among the different resistant glioblastoma cells (Fig. 1B). Genes that were differentially expressed in at least five out of six TMZ-resistant glioblastoma subclones are depicted in heatmap format (Fig. 1C). The top-6 transcripts overexpressed in all six resistant subclones were validated by qRT-PCR (Fig. 1D). EFEMP1 emerged as the most differentially expressed mRNA in all three glioblastoma cell lines with >2-fold (p<0.05) increased EFEMP1 expression in the TMZ-resistant glioblastoma subclones (Fig. 1D). Moreover, by comparing the IC_50_ values of the WT, R1, and R2 glioblastoma cell lines to the corresponding EFEMP1 expression levels, we identified a positive correlation between these two variables (r^2^=0.56, p=0.021) (Supplementary Fig. S1), suggesting that EFEMP1 expression is associated with a TMZ-resistant phenotype.

### EFEMP1 induces non-canonical TMZ-resistance in glioblastoma cells

**Figure 2:**
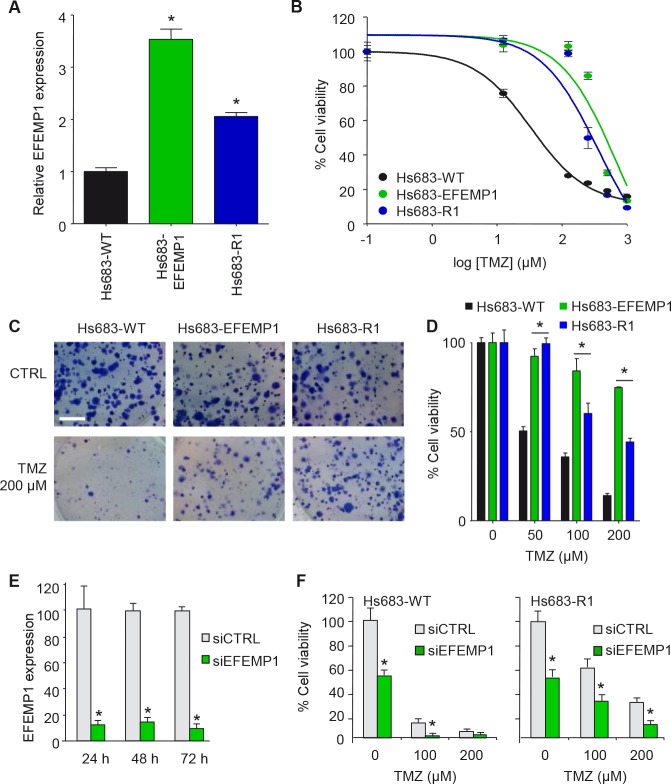
EFEMP1 induces TMZ-resistance in glioblastoma cells A, EFEMP1 mRNA expression levels of Hs683-EFEMP1, Hs683-WT and Hs683-R1 cells as determined by qRT-PCR. B, Hs683-EFEMP1, Hs683-WT, and Hs683-R1 cells treated with TMZ and cell viability measured after four days. C, representative images of the effect of EFEMP1 overexpression in Hs683 cells on TMZ sensitivity as measured by clonogenic assays. Size bar, 5 mm. D, quantification of C. E, knock down of EFEMP1 using siEFEMP1 or siCTRL in Hs683-Rl cells 48 hrs after transfection as determined by qRT-PCR. F, cell viability of cells depicted in E upon TMZ treatment as determined by a clonogenic assay. Shown are averages, error bars indicate SD. *p<0.05 t test.

To determine whether EFEMP1 can act as a sole effector of non-canonical TMZ-resistance in glioblastoma cells, we overexpressed EFEMP1 in Hs683 cells. We compared the EFEMP1 expression levels of Hs683-EFEMP1 cells to Hs683-WT and Hs683-Rl cells by qRT-PCR and showed a >3-fold upregulation of EFEMP1 in Hs683-EFEMP1 cells compared to Hs683-WT cells (Fig. 2A). We then treated Hs683-EFEMP1, Hs683-WT, and Hs683-R1 cells with TMZ, and measured cell viability four days later. The overexpression of EFEMP1 resulted in a significant increase in resistance to TMZ as compared to the parental Hs683 cells (Fig. 2B). The resistance effect of EFEMP1 overexpression in Hs683 cells was confirmed by clonogenic assays (Figs. 2C and D), which showed a significant increase in clonogenic survival of Hs683-EFEMP1 cells as compared to Hs683-WT cells. Conversely, >80% knock down (p<0.05) of EFEMP1 by siEFEMP1 as demonstrated by qRT-PCR (Fig. 2E), resulted in reduced cell survival of TMZ-resistant Hs683-Rl and Hs683-WT cells, as assessed by clonogenic analysis after TMZ treatment (Fig. 2F). However, significant effects of EFEMP1 knock down on cell survival were also observed in the absence of TMZ treatment, indicating a dependence of these cells on EFEMP1 expression for survival, as was previously described [22].

### EFEMP1 induces Notch signaling in glioblastoma cells

**Figure 3:**
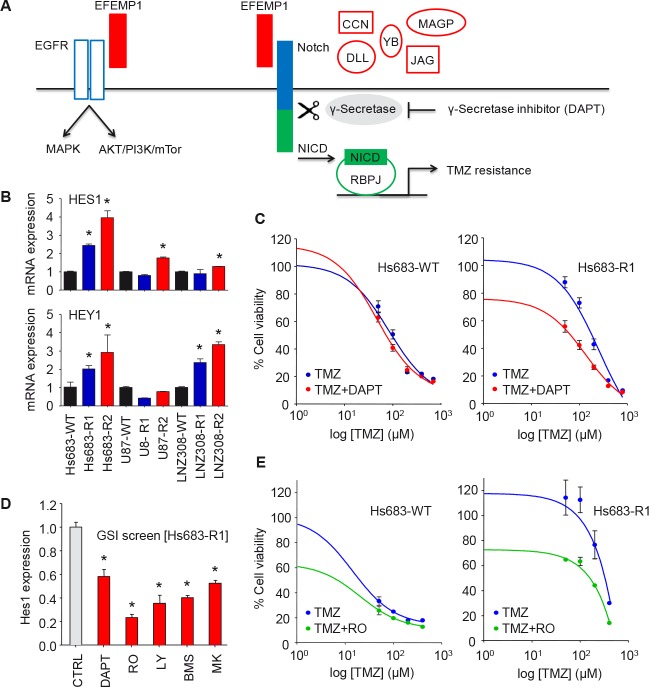
EFEMP1 induces Notch signaling in glioblastoma cells A, schematic overview of EFEMP1 activation of EGFR [19] and Notch [22] signaling. MAGP-1/2, CCN3, YB-1, DLL, and Jagged are alternative Notch ligands [46–48, 50, 51]. B, mRNA expression of the Notch-induced genes HES1 and HEY1 in the TMZ-resistant glioblastoma cells as determined by qRT-PCR. C, cell viability analysis of Hs683-WT and Hs683-Rl glioblastoma cells four days after treatment with TMZ and DAPT (25 μM). D, HES1 expression after treatment with clinically available GSIs R04929097, BMS-708163, LY450139, and MK-0752 as determined by qRT-PCR. E, cell viability analysis of Hs683-WT and Hs683-R1 four days after treatment with TMZ and RO4929097 (50 μM). Shown are averages, error bars indicate SD. *p<0.05 t test.

**Figure 4:**
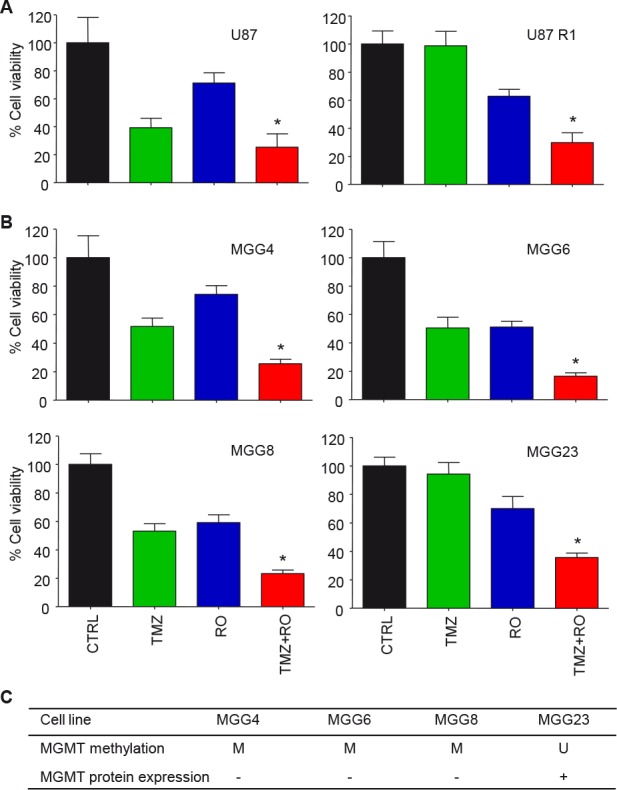
RO4929097 and TMZ treatment of glioblastoma cells in vitro A, cell viability analysis of U87-WT and U87-R1 cells four days after treatment with RO4929097 (30 μM) and TMZ (100 μM). B, similar as A for a panel of primary glioblastoma cell lines. C, MGMT methylation status and MGMT protein expression of the individual primary glioblastoma cell lines used in B. Shown are averages, error bars indicate SD. *p<0.05 t test.

EFEMP1 as part of the extracellular matrix can exert its cellular signaling function via multiple pathways, including the EGFR and Notch signaling cascades (Fig. 3A) [19, 22]. In order to determine whether EFEMP1 overexpression resulted in the activation of the Notch signaling pathway in the TMZ-resistant glioblastoma cells, we first assessed the expression of the Notch-induced genes HES1 and HEY1 by qRT-PCR (Fig. 3B). We show that HES1 and/or HEY1 were >2-fold overexpressed (p<0.05) in the TMZ-resistant cells, except for U87-R1. Subsequently, we determined whether blocking of the Notch pathway could overcome the EFEMP1-mediated resistance to TMZ, by treating the Hs683-WT and Hs683-Rl glioblastoma cells that demonstrated high expression of HES1/HEY1 with TMZ and the γ-secretase inhibitor (GSI) DAPT (Fig. 3C). Treatment of Hs683-R1 with DAPT in the absence of TMZ resulted in a 20% decrease in cell viability (p<0.05), which was not observed in Hs683-WT cells. Importantly, in combination with TMZ, treatment with DAPT resulted in enhanced cell kill as compared to TMZ alone (Fig. 3C). Subsequently, we assessed the inhibitory effects of the clinically available GSIs RO4929097, BMS-708163, LY450139, and MK-0752 on the Notch pathway by determining HES1 expression after GSI treatment (Fig. 3D). At the concentration used, treatment with RO4929097 resulted in the largest reduction (approximately 75%. p<0.05) of HES1 expression in Hs683-Rl glioblastoma cells. Therefore, we selected RO4929097 for combined treatment with TMZ on Hs683-WT and Hs683-R1 cells. Similar as for treatment with DAPT (Fig. 3C), RO4929097 treatment alone resulted in decreased cell viability and in combination with TMZ, RO4929097 treatment significantly enhanced cell kill compared to TMZ alone in the TMZ-resistant Hs683-Rl cells (Fig. 3E). In addition, we demonstrated similar effects of combined treatment with TMZ and RO4929097 on U87-WT and U87-R1 cells (Fig. 4A) and on primary glioblastoma cell lines (Fig. 4B). These primary glioblastoma cells were characterized for their MGMT promoter methylation status and MGMT protein expression (Fig. 4C). We showed that treatment with RO4929097 potentiated the TMZ effect on U87-R1 and the primary glioblastoma cell lines, including on MGG23 cells that were the only primary glioblastoma cells included to have an unmethylated MGMT promoter and high expression of MGMT. Altogether these data suggest that GSIs can decrease Notch signaling in glioblastoma cells and potentiate the TMZ effect on glioblastoma cells that acquired EFEMP1-mediated TMZ-resistance. Moreover, these results support previous reports on TMZ-sensitizing effects of GSIs in glioblastoma cells [24–26].

### Effect of RO4929097 in combination with TMZ treatment on orthotopic Hs683 glioblastoma in vivo

**Figure 5:**
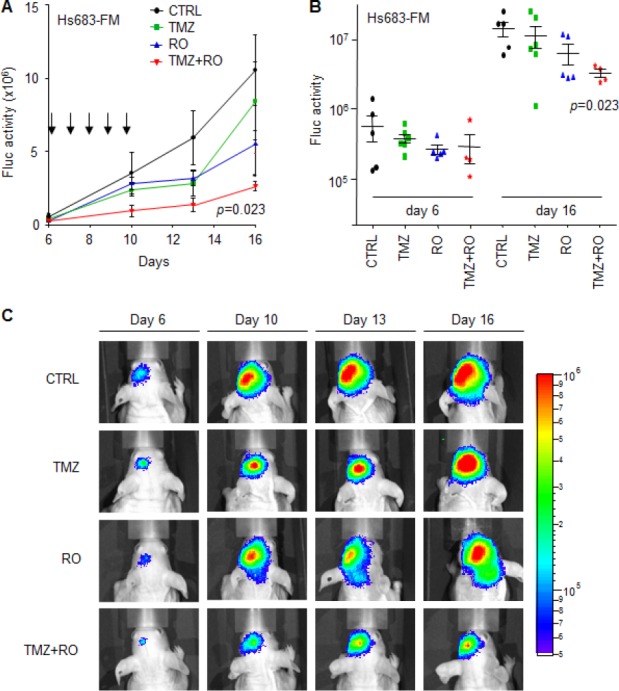
RO4929097 and TMZ treatment of orthotopic Hs683-Fluc glioblastoma in vivo A, mean Fluc activity values of different treatment groups measured over time using a CCD camera. Arrows indicate five consecutive treatment days starting at day six after Hs683-Fluc cells injection in the striatum of nude mice. B, Fluc activity values of individual mice per treatment group at the start of treatment (day 6) and six days after treatment had finished. C, representative Fluc bioluminescence images of treatment groups at different time points after injection of cells (day 0). Shown are averages, error bars indicate SEM. The p values indicate two-sided t-test.

In order to assess whether inhibition of the Notch pathway could potentiate TMZ treatment in orthotopic glioblastoma in vivo, Hs683 cells were transduced with a lentiviral vector encoding the Fluc bioluminescence reporter generating Hs683-Fluc. Next, Hs683-Fluc glioblastoma cells were injected into the striatum of nude mice (n=24). Six days post-implantation the luciferase signal was measured and mice were randomized into four treatment groups (i.e. CTRL; TMZ; RO4929097; TMZ+RO4929097; n=6 per group). Directly after randomization, mice were treated with TMZ and/or RO4929097 for five consecutive days. At day 1 and 5 of treatment, mice were treated with only RO4929097 while on day 2, 3, and 4 mice were treated with both TMZ and RO4929097 or corresponding vehicles. We assessed glioblastoma growth over time by Fluc bioluminescence imaging. Fluc activity demonstrated that treatment with TMZ+RO4929097 significantly reduced Hs683-Fluc tumor growth in vivo compared to tumor growth in non-treated mice (p=0.023) (Figs. 5A–C), although under these devastating conditions we were not able to measure significant effects on survival (survival 18±6 days after injection of the Hs683-Fluc cells). These results suggest that inhibitors of the Notch pathway may sensitize glioblastoma cells to TMZ in vivo.

### EFEMP1 expression correlates to TMZ treatment efficacy and survival in glioblastoma patients

**Figure 6:**
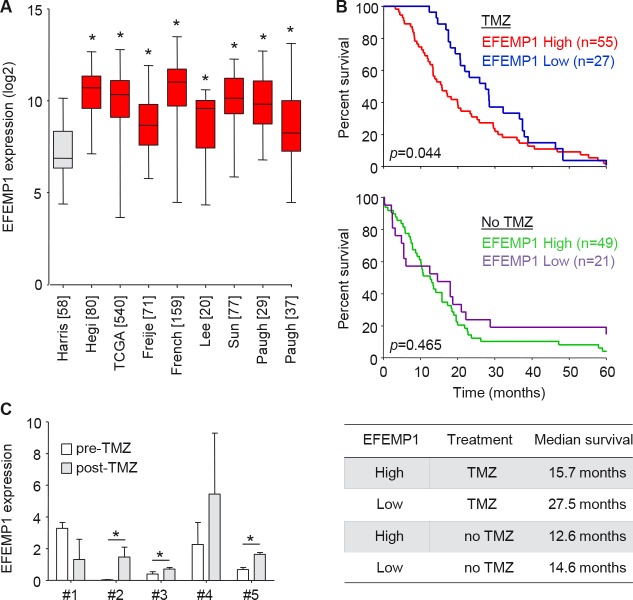
EFEMP1 expression correlates to TMZ treatment efficacy and survival in glioblastoma patients A, EFEMP1 expression levels in multiple publicly available glioblastoma datasets as visualized by using R2 (R2.amc.n1). Glioblastoma datasets (red) [28–34] were compared to normal brain (grey) [27]. [Number of patients] included in dataset are shown. B, survival of glioblastoma patients with high- (red and green), or low (blue and purple) EFEMP1 expression was plotted for TMZ-treated (n=82, upper panel) or non-treated (n=70, lower panel) patients (www.rembrandt.org). C, EFEMP1 mRNA expression levels in patient samples upon clinical progression following TMZ treatment (post-TMZ), compared to matching samples biopsied at diagnosis (pre-TMZ). Numbers (#) represent individual glioblastoma patients. Expression levels were normalized to expression in non-neoplastic brain tissue. Shown are averages, error bars indicate SD. *p<0.05 t test.

In order to determine the clinical relevance of EFEMP1 in glioblastoma, we determined the EFEMP1 expression levels in multiple publicly available glioblastoma datasets [27–34] using the microarray analysis and visualization platform R2 (http://r2.amc.n1) and showed a significant overexpression of EFEMP1 in glioblastoma tissues as compared to non-neoplastic brain tissue (Fig. 6A). Next we analyzed whether EFEMP1 expression is a predictive biomarker candidate for the efficacy of TMZ treatment in glioblastoma patients. Therefore, we analyzed overall survival of glioblastoma patients treated with or without TMZ (TMZ-treated n=82, not TMZ-treated n=70, www.rembrandt.org) in correlation with EFEMP1 expression. When EFEMP1 expression was high (>2-fold increased compared to non-neoplastic tissue, red and green line), patients had a worse prognosis in the TMZ-treated group (Fig. 6B, upper panel), while no difference in survival was observed in the non-TMZ treated group (Fig. 6B, lower panel). Correspondingly, patients with low EFEMP1 expression (<2-fold increased compared to non-neoplastic tissue, blue and purple line) had a relatively good prognosis when treated with TMZ (Fig. 6B). Thus, EFEMP1 is a significant biomarker candidate for survival in the TMZ-treated group (p=0.044), but not in glioblastoma patients that are not treated with TMZ (p=0.46). These results suggest that EFEMP1 overexpression is predictive for the resistance to TMZ in glioblastoma patients. Finally, we determined if EFEMP1 expression levels had increased in patient samples upon progression of disease following TMZ treatment. Therefore, EFEMP1 mRNA expression was determined by qRT-PCR in relapse samples and compared to their expression in the corresponding samples at diagnosis. Although we had a limited number of samples, a clear trend could be observed towards an increased expression of EFEMP1 in glioblastoma samples at the time of progression (Fig. 6C), suggesting that EFEMP1 could be used as a marker for tumor progression after TMZ treatment.

## DISCUSSION

Glioblastoma is the most common malignant primary brain tumor with a median survival rate of 14 months. While adding TMZ to radiotherapy results in an overall increase in survival of glioblastoma patients, almost all patients die. Multiple TMZ-resistance mechanisms have been demonstrated, including activity of MGMT [8, 9], MMR [14, 15], but also BER resistance mechanisms [35–37], and drag efflux pumps [38]. However, it is unlikely that these mechanisms are responsible for all TMZ-resistance observed. Here we attempted to determine non-canonical factors that may influence the efficacy of TMZ in glioblastoma. Using gene expression analysis of both TMZ-sensitive and TMZ-resistant glioblastoma cell lines, we demonstrated that overexpression of EFEMP1 is associated with a TMZ-resistant phenotype in glioblastoma cells.

EFEMP1 (Fibulin-3) belongs to the family of fibulin proteins, which are secreted proteins that integrate into the extracellular matrix. These proteins form anchoring structures that were reported to regulate cell proliferation, adhesion, and migration [39, 40], and several fibulins have been associated with the development of solid tumors [39, 40]. EFEMP1 is not significantly expressed in normal brain [21, 27, 41, 42], however, it is highly expressed in high-grade gliomas, where it promotes tumor growth and invasion [21, 22]. Glioblastomas consist of different molecular subtypes, i.e. classical, mesenchymal, neural, and proneural glioblastoma subtypes [43]. It was previously described that EFEMP1 is a mesenchymal-related gene highly expressed in primary brain tumors [21, 44]. In addition, EFEMP1 levels were higher in gliomas of the mesenchymal subtype compared to the proneural subtype which have poor overall survival compared to the other subtypes [22]. It would be of interest to determine whether EFEMP1 expression correlates with TMZ resistance and survival in this glioblastoma subclass. In contrast, a recent report [45] identified patients with glioblastomas of the proneural subtype enriched as responders to Notch inhibition via y-secretase inhibitors. These proneural glioblastomas were characterized by high Notch pathway activation. Yet unknown is whether these patients also have increased levels of EFEMP1 expression.

Here we demonstrate a significant role for EFEMP1 in TMZ-resistance of glioblastoma cells. At this point, however, inhibitors of EFEMP1 are not yet available. EFEMP1 can act on multiple pathways, including EGFR [19], and the downstream pathways MAPK [19], and AKT/PI3K/mTor [19, 20, 23]. Moreover, EFEMP1 has been identified as an activator of Notch signaling which results in increased invasion, tumor cell self-renewal, chemoresistance, and glioblastoma growth [22]. It was postulated that EFEMP1 can serve as a target that restricts anti-invasive and chemosensitizing effects to tumor cells without the pleiotropic effects of current pharmacologic inhibitors of Notch [22]. In our study, we show that EFEMP1 overexpression activates the Notch pathway in TMZ-resistant glioblastoma, while interference with the Notch pathway conveys partial sensitivity to TMZ-resistant glioblastoma cells. It should be noted that Notch signaling can also be activated in an EFEMP1-independent manner. Therefore, it remains to be determined what the exact contribution of EFEMP1 to Notch signaling is in TMZ-resistant glioblastoma cells in relation to other factors that can induce Notch signaling, including MAGP-1/2, CCN3, YB-1, DLL, and Jagged [46–51]. Furthermore, as mentioned above, EFEMP1 can act on other pathways, including EGFR which could also convey resistance to glioblastoma cells and could explain that only partial sensitization to TMZ treatment is observed upon Notch inhibition. Considering this, it would be of interest to examine the potential involvement of these pathways in providing TMZ-resistance.

Although we were able to demonstrate significant effects of RO4929097 on TMZ sensitivity in vitro, and as measured by a reduction of orthotopic Hs683-Fluc tumor growth in mice in vivo, we were not able to measure significant effects on survival between the different treatment groups, which we mainly attribute due to the relatively large variation in survival upon growth of these devastating orthotopic Hs683-Fluc tumors in the brains of mice. Supportive of our results are the previous reports on Notch pathway inhibitors synergizing with TMZ treatment in other glioblastoma models [24–26]. And, importantly, the first clinical trial of the GSI RO4929097 with TMZ treatment in glioblastoma patients has been initiated (http://clinicaltrials.gov/show/NCT01119599). It would be of interest to determine whether EFEMP1 expression levels correlate with the effect of GSIs and TMZ on patient survival in this patient group. Moreover, blood plasma-based assays are available to quantify secreted EFEMP1 expression levels [52]. Further research is needed to determine whether EFEMP1 level in glioblastoma tissue and/or in blood could serve as a stratification marker for the treatment of patients with TMZ and/or GSIs.

In conclusion, we provide evidence for a role of EFEMP1 in TMZ-resistance in glioblastoma. Inhibition of EFEMP1 and/or its downstream signaling components may be a potential therapeutic venue for the sensitization of glioblastoma cells to TMZ.

## MATERIALS AND METHODS

### Cells

Human glioblastoma cell lines Hs683, U87, and LNZ308 were cultured in DMEM (PAA Laboratories, GmbH, Pasching, Austria) supplemented with 10% fetal bovine serum (FBS) (PAA Laboratories, GmbH, Pasching, Austria), and 1 mg/ml penicillin-streptomycin (both PAA Laboratories, GmbH, Pasching, Austria) at 37°C and 5% CO2 in a humidified incubator. TMZ (Schering Plough, Kenilworth, NJ, USA) was dissolved in dimethyl sulfoxide (DMSO) to prepare a stock concentration of 150 mM which was further diluted in cell culture medium to the working concentration. To generate TMZ-resistant cell lines, parental cells were treated twice a week in duplicate with a clinically relevant concentration of TMZ (33 μM). Exposure to TMZ was continued for multiple weeks (10– 18 weeks), until two individual resistant subclones were generated of each parental glioblastoma cell line. TMZ-resistant cells were regularly challenged with 33 μM TMZ to maintain the resistant phenotype. Passage controls which were not treated with TMZ were cultured in parallel to use as controls for further analysis. To generate Hs683 cells overexpressing EFEMP1, cells were transfected with an EFEMP1-overexpression construct kindly provided by Dr. M.S. Viapiano (The Ohio State University Medical Center and James Comprehensive Cancer Center, Columbus, OH, USA). Transfected glioblastoma cells were selected using 250 μg/ml of Zeocin Selection Reagent (Invitrogen, Carlsbad, CA, USA). For in vivo experiments, Hs683 cells were stably transduced with a lentivirus vector encoding firefly luciferase (Fluc) [53] to establish Hs683-Fluc cells. Primary glioblastoma cells, MGG4, MGG6, MGG8, and MGG23 were kindly provided by Hiroaki Wakimoto (Massachusetts General Hospital, Boston, MA, USA), and cultured as previously described [54]. The cells used in this study were not authenticated.

### Gene expression profiling

Gene expression profiling was performed at the VUmc array core facility as described elsewhere [55, 56]. Briefly, RNA samples were Cy3- or Cy5-labeled using the Agilent Low RNA Input Linear Amplification Kit Plus, according to the manufacturer's protocol. Subsequently, equal amounts (825 ng) of the Cy3 and Cy5 labeled samples were hybridized to Agilent 4×44K Whole Human GE arrays (Agilent), according to the manufacturer's instructions. Samples derived from each individual glioblastoma cell line (both parental cell line and resistant subclones) were hybridized on a single slide. Slides were scanned using an Agilent Microarray scanner (G2565BA). Image analysis was performed using feature extraction software version 9.5 (Agilent Technologies). The Agilent GE2-v5_95 protocol was applied using default settings. Array normalization was performed with R-package Limma within the Bioconductor software. The microarray data have been deposited in NCBI's Gene Expression Omnibus (GEO, http://www.ncbi.nlm.nih.gov/geo), Series Accession Number GSE53014.

### qRT-PCR

Quantitive RT-PCR (qRT-PCR) analysis was used to determine the relative expression levels of EFEMP1, HES1, HEY1, and GAPDH mRNA. Total RNA from cell lines was isolated using TRIzol RNA isolation protocol (Invitrogen, Carlsbad, CA, USA) and total RNA from paraffin-embedded glioblastoma tissue was isolated using the RecoverAllTM Total Nucleic Acid Isolation Kit (Ambion, USA). Equal amounts of RNA were converted into cDNA using Omniscript kit (Qiagen, Hilden, Germany). Subsequently, qRT-PCR was performed with primers designed using Primer-Blast (NCBI) and manufactured by Invitrogen. The Ct values were used to calculate the relative fold difference in mRNA levels. The data were normalized to GAPDH expression levels.

### Cell viability/survival assays

Cells were plated in 96-well plates and treated the subsequent day with increasing concentrations of TMZ and/or the γ-secretase inhibitors RO4929097, DAPT, BMS-708163, LY450139, MK-0752 (SelleckChem, Houston, TX, USA). Four days after treatment, cells were fixed with 3.7% formaldehyde and DNA was stained with DAPI diluted in PBS (0.3 μg/ml). Cell number was assessed by counting the number of DAPI-stained cells using the Acumen Ex3 laser scanning cytometer (TTP LabTech, Royston, UK). Clonogenic assay was performed by plating single cells in 6-well plates. Three different cell dilutions (each in duplicate) were used per plate. For siRNA experiments, cells were cultured in a 6-well plate, transfected after 24 hrs with 66 nM of siEFEMP1 or siCTRL using lipofectamine 2000 (Invitrogen, Carlsbad, CA, USA), and plated as single cells subsequent day in three different cell dilutions. The next day, cells were treated with different drag concentrations. After 11–14 days, colonies were washed with PBS, fixed with 3.7% formaldehyde, stained with Giemsa staining (1:10 in demi water), washed with water to remove the excess of Giemsa, and air dried. The number of colonies was determined by marking the stained colonies containing >50 cells using light microscopy. The survival fraction was determined by normalizing the number of colonies formed in the treatment condition to the number of colonies formed in the control condition [number of colonies formed/(number of cells plated × plating efficiency)]. Plating efficiency is defined as the percentage of cells plated that actually formed colonies in the control group.

### In vivo experiments

All animal studies were approved by the institutional ethical committee on animal experiments of the VU University (Amsterdam, The Netherlands). Six-weeks old female athymic nude mice (Harlan Laboratories, Horst, The Netherlands) were kept under specific-pathogen free conditions in air-filtered cages and received water and food ad libitum. Five μ1 containing 5×10^5^ Hs683-Fluc cells was stereotactically injected into the striatum of nude mice (coordinates from bregma: −2.0 mm X, +0.5 mm Y, −3.0 mm Z). Tumor size was monitored by Fluc bioluminescence imaging twice per week. At six days post-injection of cells, mice were divided into four treatment groups (n=6 per group). The control group received intraperitoneal (i.p.) injection of 200 μ1 saline (TMZ-vehicle), and/or per os (p.o.) injection of 200 μ1 0.5% methylcellulose in sterile water (RO4929097-vehicle). TMZ-only treated group received 10 mg/kg TMZ i.p. and vehicle p.o., while the RO4929097-treated group received 30 mg/kg RO4929097 p.o. and vehicle i.p.. The combined treatment group was injected with the same concentration of TMZ i.p. and RO4929097 orally. Mice were treated once daily for five consecutive days with RO4929097 or vehicle and on day 2, 3, and 4 with TMZ or vehicle. Mice were sacrificed after the humane endpoints were reached via sedation using isoflurane followed by i.p. injection of Euthasol. To assess significant differences between treatment groups, mice with Fluc bioluminescence values within each treatment group outside the mean ±2 × SD range were excluded from further analysis.

### Statistical analysis

Difference in biological properties between treated and untreated cells was analysed using two-sided Student's t-test. The p values <0.05 were considered statistically significant. SAM analysis (false discovery rate <10%) was used to determine significantly differential expressed genes in the gene expression microarray data [57]. Pearson's correlation was used to determine association between IC^50^ values and EFEMP1 mRNA expression. Survival curves were compared by log-rank test.

## Supplementary Figure and Table




