# Supplementary material for: EFEMP1 induces γ-secretase/Notch-mediated temozolomide resistance in glioblastoma
Source: Oncotarget. 2013 Dec 7;5(2):363–74. doi: 10.18632/oncotarget.1620 (PMC3964213; doi:10.18632/oncotarget.1620)
Supplement: Supplementary file 1 [file oncotarget-05-363-s001.pdf]

EFEMP1 induces γ-secretase/Notch-mediated temozolomide resistance in glioblastoma – Hiddingh et al

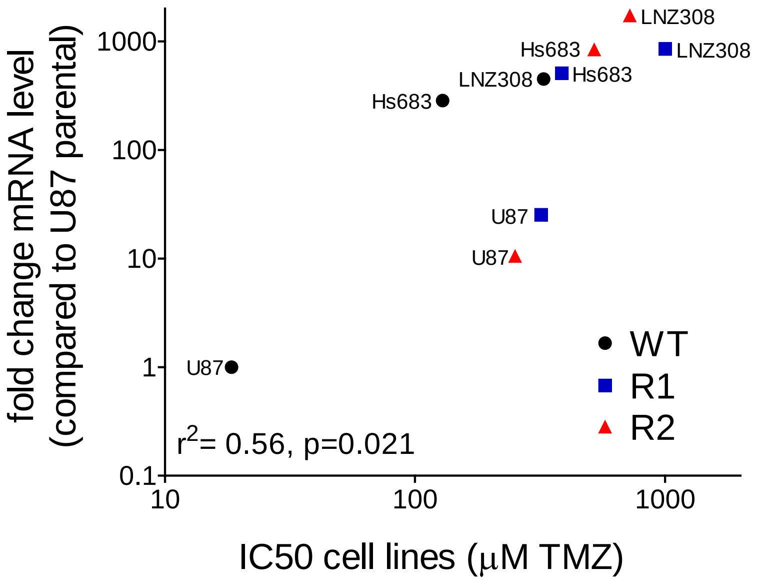

Supplementary Figure S1: Correlation of TMZ IC_{50} values and EFEMP1 mRNA expression levels. IC_{50} values of the WT, R1, and R2 glioblastoma cell lines were compared to the corresponding EFEMP1 expression levels (r^2=0.56 p=0.021).
