# Supplementary material for: EFEMP1 induces γ-secretase/Notch-mediated temozolomide resistance in glioblastoma
Source: Oncotarget. 2013 Dec 7;5(2):363–74. doi: 10.18632/oncotarget.1620 (PMC3964213; doi:10.18632/oncotarget.1620)
Supplement: Supplementary file 2 [file oncotarget-05-363-s002.pdf]

EFEMP1 induces γ-secretase/Notch-mediated temozolomide resistance in glioblastoma – Hiddingh et al

Supplementary Table S1: Characteristics of the parental and TMZ-resistant (R1 and R2) glioblastoma cell lines. IC_{50} values, MGMT methylation index, microsatellite instability and MSH6 mutation status of the individual cell lines are shown. MGMT methylation index was determined by MLPA (multiplex ligation-dependent probe amplification).

Cell line		IC_{50} (μM TMZ)	MGMT methylation index	Microsatellite instability	MSH6 mutation
Hs683	Parental	128.9	0.5	No	No
	R1	386.4	0.3	No	No
	R2	520.5	0.3	No	No
U87	Parental	18.45	0.8	No	No
	R1	319.5	1.0	No	No
	R2	251.3	0.8	No	No
LNZ308	Parental	326.7	0.8	No	No
	R1	1001.0	0.7	No	No
	R2	723.5	0.8	No	No
